# Unmet Needs and Classical Pitfalls in the Management of Adolescents With Behavioral Problems in Emergency

**DOI:** 10.3389/fpsyt.2021.527569

**Published:** 2021-02-12

**Authors:** Marie-Jeanne Guedj-Bourdiau, Jean-Marc Guilé, Sébastien Garny de la Rivière, Ugo Pace, David Cohen, Xavier Benarous

**Affiliations:** ^1^Centre Psychiatrique d'Orientation et d'Accueil, Hôpital Sainte Anne, Paris, France; ^2^Department of Child and Adolescent Psychopathology, Amiens University Hospital, Amiens, France; ^3^INSERM Unit U1105 Research Group for Analysis of the Multimodal Cerebral Function, University of Picardy Jules Verne (UPJV), Amiens, France; ^4^Department of Psychiatry, McGill University, Montreal, QC, Canada; ^5^Etablissement Public de Santé Mental de la Somme, Amiens, France; ^6^Department of Child and Adolescent Psychiatry, Pitié-Salpêtrière Hospital, Paris, France; ^7^CNRS UMR 7222, Institute for Intelligent Systems and Robotics, Paris, France

**Keywords:** aggressive behavior, behavioral problem, emergency, crisis-intervention, emergency department, adolescent

## Abstract

While behavioral problems are the main reasons for adolescents to be referred to an emergency room for mental health problems, their clinical management remain usually heterogenous, poorly standardized, and associated with a low level of patient and family satisfaction. So far, most attention has been paid to the treatment of agitation, and few insights have been provided on the treatment plan of behavioral problems once the crisis is over and a psychiatric or medical condition ruled out. This perspective article represents an attempt to incorporate multiple theoretical approaches to provide a comprehensive and operational model for the management of adolescents with behavioral problems in an emergency department. Short hypothetical case vignettes illustrate the importance of considering several levels of analysis to understand the adolescent's problematic behavior which can be seen as a symptom of a medical/psychiatric condition (medical model), as a maladaptive strategy in a context of vulnerability (developmental model), and finally as a mode of communication in a context of ill-adapted relational patterns (systemic model). As behavioral problems in adolescence are a complex issue, frequently involving the intervention of professionals from various disciplines, being aware of such different levels of understanding could help to preclude any role confusion and to provide better targeted interventions.

## Introduction

Over the past 15 years, the number of pediatric patients presenting to the emergency room for mental health problems has nearly doubled (1). Behavioral problems are the main reasons for adolescents to be referred to a psychiatric consultation in Pediatric Emergency Department (PED), accounting for 11–55% of all mental health visits (1–3). Heightened awareness of mental health problem in adolescent and the lack of available community resources in most regions has been incriminated to explain this surge (4). In addition, such visits for mental health problems in PED are in most cases the adolescent's first point of contact with a mental health care system (with 53–70% of youths without prior outpatient care) (5–7).

Several authors stressed that a lack of specific training for adolescent psychiatry in PED results in heterogeneous care and low levels of both patient and family satisfaction, in particular due to excessively long wait times (3). In particular, many professionals in PED may feel uncomfortable in caring for adolescents with behavioral problems hastily perceived as not having a “real” urgent medical problem or whose presence in a noisy crowded emergency room is deemed irrelevant or even dangerous for other patients. When the clinician in charge of the psychiatric assessment of such patients in PED is alone, lacking time or having no specific interest in adolescent psychiatry, his/her evaluation might be limited to the following question: “*Has my young patient an acute psychiatric disorder requiring urgent medication or inpatient care?*” Albeit essential, a positive answer only concerns a minority of adolescents with behavioral problems addressed to PED and thus it is not clear what to do with others. A longitudinal study including a systematic assessment of acuity rating for all patients in PED showed that the proportion of non-urgent mental-health related visits increased by 31% between 2003 and 2012 (8). Letting these adolescents go home without any other response certainly miss important needs, paving the way for future referrals.

This perspective article represents an attempt to integrate multiple theoretical approaches of adolescent behavioral problems to provide a series of practical advices for all professionals involved in their care.

## Definitions

Behavioral problems in adolescence is not a homogeneous category. It encompasses a wide array of problematic behaviors ranging from oppositions, aggressive behavior, to antisocial behavior such as theft or runaway from home (9). Some clinical dimensions associated with behavioral problems in adolescents, such as impulsivity or irritability, are continuously distributed in the general population with those at one extreme of the spectrum having a high risk of associated psychiatric disorders. However, adolescents with psychiatric disorders represent only a small proportion of all teenagers with behavioral problems. Adolescent's behavioral problems directly imputable to psychiatric symptoms such as severe anxiety, delusion or dissociation are in fact rather the exception than the rule. In theory, in these cases the adolescent should no longer present any behavioral problems when his/her mental health problems are addressed. In many of cases, the situation are more complex and the adolescent's behavioral problems reflect maladaptive reactions associated with environmental and individual factors that would persist after the ED visit (10, 11). However, while the guidelines for the management of behavioral problems in PED (12, 13) pay particular attention to the treatment of agitation, few insights are provided on the treatment plan of behavioral problems once the crisis is over and a psychiatric or medical condition ruled out.

The term emergency is generally defined as a sudden and dangerous situation which needs immediate action to deal with it (14, 15). The concept can also be approached from a functional perspective, i.e., each presentation at an emergency service should initially be viewed as an emergency (16). Using this second definition, the key question for the clinician is no longer “*Are the reasons for referral of this teenager valid on my medical/psychiatric view?*” but rather “*what dangerous situations were feared by the adolescent and his surroundings and what kind of urgent interventions were expected to motivate the referral in emergency*.” Behavioral problems in adolescents that are not directly caused by a somatic condition or psychiatric symptoms generally consist of outward expressions of relational problems which may be inherent to common aspects of developmental changes in teenagers (17). While a reasonable level of indirect aggressive behavior or defiant attitude in adolescents have been viewed positively as a way for the youths to express their own subjectivity and values (17), it has also been shown that such reactions may tend to perpetuate by themselves through the reaction of the environment and the deterioration of the self-image. It can be postulated that for adolescents with behavioral problems and their surroundings, “emergency” represents the only way out of a persisting relational crisis. In such crises the adolescent and his family lack the internal or external resources to bring about an attitudinal change or, if necessary, a change of the interpersonal setting to find a new balance.

## Understanding Unmet Needs of Adolescents With Behavioral Problems and Their Surroundings

In this section, we present a hierarchical three-step model for the assessment of adolescents with behavioral problems in PED (Table 1). The steps differ in terms of principal aims, level and object of analysis, and the underlying theoretical model. We hypothesized that the low level of a patient's and family's satisfaction in a context of referral to PED for the adolescent's behavioral problems partly result from a discrepancy between the level of the patient's and the family's expectancies and the responses provided by the care system.

**Table 1:** A proposed three-step approach for the assessment of adolescents with behavioral problems in ED.

**Step**	**1 Medical/psychiatric assessment**	**2 Developmental hypothesis**	**3 Systemic formulation**
Emergency model	Triage model	Formulation model	Crisis intervention model
Underlying theoretical model	Medical model, i.e., diagnosis, prognosis, and treatment	Developmental psychology	Time-limited psychotherapeutic approach, systemic model
Level of analysis	Adolescent patient	Adolescent and his caregivers	Adolescent within his system
Object of analysis	Psychiatric symptoms	Adaptative and maladaptive emotional and behavioral strategies and family resources	Repetitive relational patterns
Principal aims	Medical decision (assessment, treatment, referral, sometimes short follow-up)	Providing information and guidance	Consultative roles

The first step of the assessment of adolescents referred to PED for behavioral problems consists in basic medical and psychiatric assessment. The clinical assessment has three objectives: (1) to screen out a psychiatric disorder, a toxic or medical condition that may cause the problematic behavior, (2) to estimate the risk of harm to self or others, and (3) to determine the need for prescription and/or an inpatient treatment. This step is brief and should focus on the determination of priority for treatment (for an illustration see Table 2, case 1).

**Table 2:** Hypothetical case vignettes synthesized from several real cases of adolescents referred to PED for aggressive behavior.

**Clinical vignette 1**
M., a 16-year-old boy, was rushed to the hospital for extreme agitation by a social educator. He had lived in foster care placement since he was expelled from his father's house at the age of 6 for unknow reasons. M. had no prior psychiatric or somatic history. He had had a prior visit to PED a few months earlier but ran away before the clinical assessment. The psychiatric assessment revealed severe delusional and paranoid thoughts which gave rise to feelings of hostility. He was deeply convinced of being victim of an injustice in his receiving home. The routine somatic and paraclinical investigations were normal, including toxicologic evaluation. An antipsychotic treatment with sedative effect was prescribed in PED, and the patient was addressed to a psychiatric pediatric inpatient unit for continuation of care. No physical restraint was used. The probabilistic diagnostic at the discharge of the PED was an acute psychotic episode
**Clinical vignette 2**
L., a 14-year-old girl, was brought to emergency consultation by her parents for severe aggressive behaviors against them. She is a single girl adopted 3 years previously with her biological family still living in Colombia. She had had few psychiatric consultations at age 11 for an anger-management issue. The parents reported that over the last days L. had a resurgence of the same problematic behaviors as at age 11 (mainly irritable and touchy mood) accompanied by new symptoms (secondary enuresis, loss of interest for school and leisure, and marked social withdrawn). In the emergency room, L. is initially mute, still, huddled up. After repeated consultations, alone and with the parents, she reported a first sexual relation with ambivalent consent 2 weeks earlier. Family consultations helped L. and her parents to make sense of her behavior in a context of preexisting difficulties for sharing emotional distress. We also discussed L.'s reluctance to access ambulatory care despite several emergency consultations (about 10 during the past 2 years). As sharing her inner feeling with adults is specifically her core problem, we discussed alternative treatments (family sessions, physical therapy). The probabilistic diagnosis at the discharge of the ED was adjustment disorder with depressive symptoms in a context of prior reactive attachment disorder
**Clinical vignette 3**
S., a 16-year-old boy, was brought to PED by a social educator after he threatened to strangle another adult. He had been having a psychiatric follow-up since the age of seven and had received the diagnoses of attention deficit disorder, conduct disorder, post-traumatic stress disorder, and borderline intellectual functioning. He had moved into a new foster care 2 months previously with difficulties adjusting to this new environment, with in particular a history of bullying with sexual assault of other residents, both as victim and as perpetrator. He is described as becoming notably more irritable and hostile over the past few days, staying in his room alone, watching television, and avoiding any social contacts. A contract had been formalized with his consulting psychiatrist to plan a hospitalization in the weeks to come. During clinical assessment aggressive behaviors are often minimized with a tendency to place the blame on others. A phone call to his referent educator told us much about the current situation. Following S.'s temper outbursts several adults were frightened of him, avoiding interactions or minimizing educational requirements, with episodic sudden coercive reactions by threating him of being hospitalized. The patient was also not permitted to phone-call his biological family for vague reasons (in response to his behavior or for a problem of administrative authorization). In this context of escalade of retaliatory measures between the adolescent and the educative team, a very short period of separation (12 h) was helpful to gain insight on the systemic contribution of S.'s problematic behaviors and suggest calming measures. In collaborations with a broad array of partners we suggested that at the discharge of the PED, S. might be oriented to a farmhouse for a break before attending to his inpatient admission. The educators were interested to find out how they could develop more consistent educative responses to his transgressive behaviors

Once the causal role of a somatic condition or a psychiatric disorder in the adolescent's behavioral problems are ruled out, the clinicians may elicit the developmental factors involved in the initiation or the maintenance of the problematic behaviors. In this approach, the clinician pays particular attention to the course of the psychiatric symptom/problematic behaviors, in particular in environmental maintenance factors. In this step, the clinician formulates behavioral problems in terms of disturbances of developmental processes where symptoms are regarded as maladaptive, blocked, regressive or anti-developmental behaviors (for an illustration see Table 2, case 2).

In some situations, the assessment of the adolescent's behavioral problems requires the use of a broad lens to assess the entire system involved with the adolescent. Systemic formulation of the adolescent's behavioral problems aims to better understand the repetition of stereotyped patterns of interaction between members of the system. This approach is therefore particularly useful to analyze recurrent use of PED for mental-health problems. In this step, the clinician formulates problematic behaviors in terms of communication strategies in a specific system. Here, the adolescent's behavioral problems are not regarded as a sign of malfunction (step 1) or as a maladaptive strategy (step 2), but rather as the least bad option for the teenager to maintain a specific balance in his relational system (for an illustration see Table 2, case 3).

## Classical Pitfalls in Treating Adolescent With Behavioral Problems in Emergency

In the following section, we develop a series of recommendations for clinicians involved in the management of adolescents with behavioral problems. These clinical pitfalls also illustrate the fact that correctly addressing these different dimensions involved in the adolescent's problematic behavior may be complex.

### Being a Mediator but Not Referee

**Context:** Sometimes the adolescent and his/her caregiver give radically different descriptions of the situations. Traditionally, the adult's view is systematically balanced by the statement of the adolescent who minimizes or denies implication in problematic behaviors, giving an impression of a “ping-pong” type discussion.

**Principles:** Clinicians should be careful to not take the side of either the adolescent or the caregiver in this situation. The following prerequisites should be met to help the clinician to have a role of mediator between the two parties and to avoid pointless and time-consuming arguments between the adolescent and his parents.

- Clinicians should be cautious to not try to summarize or synthetize the views of the parents of the adolescent too quickly. The different points of view should be respected and recognized positively as a more adaptative way of expressing conflictual views compared to behavioral manifestations.
- Expression of empathy by the clinician should not be a way to gain an adolescent's attention or an artificial level of trust. The time spent to understand the situations and to provide consistent and detailed explanations of the patient's mental health issue and treatment plan is the best mark of compassionate care (18).
- Clinicians should try to have adolescents and adults express conflicting views of the same situations. The expression of ambivalent emotions and cognitive dissonances represent an opportunity for all family members to take different perspectives and of getting out of Manichean views.

**Why this is not easy?** In all our medical training we are taught to consider information discrepancies as a source of bias and error. To promote the expression of ambivalent thoughts requires that the clinician accepts that one cannot understand every aspect of the situation, which is far from our traditional medical approach.

### Psychiatric Diagnoses Do Not Have the Same Meaning for Everyone

**Context:** Sometimes the question of a severe psychiatric disorder (usually schizophrenia or bipolar disorder) is raised by the caregivers while no obvious clue supports this view.

**Principles:** Confusion may exist in the caregivers' perceptions of the severity of the situation in terms of overall functioning and in terms of clinical severity. The severity of the problematic behavior due to its impact on the physical health (e.g., due to physical injury) or social consequence (e.g., family conflict, being expelled from school) is not necessarily correlated with the psychiatric severity. Family and caregivers can press for this diagnosis when it represents an opportunity for hospitalization and thus a separation. Of course, the description of the adolescent's behavior may be partly affected by specific concerns about a psychiatric disorder due, for example, to family history (for example normal mood swings is described as “pathological mood lability”).

- We have to accept that sometimes the diagnostic formulation is impossible in emergency consultations, considering the lack of time, objective information about prior functioning, and the difficulties to conduct a clinical assessment when the adolescent is reluctant or outrightly hostile. No diagnosis (with a focus on clinical syndrome or a probabilistic diagnostic) is always a better option than a bad diagnosis. An excessive focus on the most salient behavioral problems may lead to an overdiagnosis of externalizing disorders or borderline personality disorder.
- Sometimes comorbid psychiatric disorder may be essential to consider for risk assessment. For example, in youths with conduct disorder and psychopathic traits, depressive symptoms, albeit rare, may have dramatic consequences with a very high risk of impulsive suicidal attempt.
- For the clinicians the diagnosis is a way to choose the most-appropriate intervention; however, for the family it is expected to shed light on the motivations behind a behavior viewed as aberrant, chaotic and problematic. For most adolescents with behavioral problems the diagnosis is an adult way to talk about things that they truly do not understand.

**Why this is not easy?** Diagnoses are part of our medical identity; coding diagnosis is also an obligation in most countries to account for our clinical activity in ED.

### Being Cautious When There Is an Excessive Emphasis on External Factors

**Context:** Sometimes the adolescent and his caregivers extensively discuss during the consultation about a stressful life event, generally occurring outside the family (e.g., peer-abuse, medical problem, potentially traumatic events in childhood).

**Principles:** Some environmental risk factors seem more “concensual” to incriminate than others. In particular, all life events that do not involve one of the persons present in the PED, such as school teachers, a girlfriend, or biological family in a context of foster care. Albeit important to consider, such biographical events should not hide other factors potentially relevant *hic* and *nun* to understand the maintenance of the problematic behaviors. A high level of parental shame about the adolescent's behavioral difficulties may explain that some of these adults are reluctant to talk about what has happened inside the family. External factors could appear as consensual topics of discussion toward which they can turn their blame.

- Above all, it is essential to carefully assess adverse life events when they are mentioned by the family.
- It is preferable to limit the discussion to one or two life events rather than trying to superficially address too many things. To go to the depths means to adopt active listening by making clear facts (*what has happened next?*), feeling (*what did you feel?*), thoughts (*why did you feel that way?*), reactions (*how did you react?*), and expected reactions from the surroundings.
- When parents and adolescents are both reluctant to talk about their own authentic feelings, you can try to get them to discuss about someone else's perceptions (“*how do you expect your parent to behave at this moment? Why?*”). Sometimes adolescents lacking insight on their own emotions may be surprisingly very good at acknowledging a feeling of hopelessness or shame conveyed by one of the parents. Such an intersubjective game should be practiced with the patient and the parents together.

**Why this is not easy?** Families may have the feeling that you are trying to find new problems. This resistance reflects how afraid they could be of moving from a stability stance even if this means partially denying the adolescent's problem inside the family.

### Is All About Control

**Context:** All of your suggestions, even the most paradoxical, are rejected by the adolescent. The more you suggest positive solutions, the closer the adolescent gets to discussion.

**Principles:** At this point, the clinician should keep in mind that behavioral problems represent for many the least bad options vulnerable adolescents have to maintain a sense of control and predictability in their life. Helping to gain a sense of control requires letting the patient have the possibility to address his needs in a different time and a different context. Accepting help from mental health professionals means challenging stigma about mental health problems. All of these may be particularly difficult for adolescents whose attachment issues make them less prone to trust adults and to commit to a therapeutic relationship.

- Talking about resistance and possible stigma in an open way may be more useful than trying to convince an adolescent to accept an intervention (e.g., medication, new referral).
- Doctors are often seen as figures of authority, and “recommendations” may be perceived as being directive or giving an order. Nurse specialists are often considered to be less authoritative, so discussions with them or other non-medical professionals may be worthwhile in an emergency so as not to be confined to a duel scenario, repeating a coercive relational pattern.
- Sometimes it helps to remember that our long-term goals are to decrease stigma about mental health difficulties and help these adolescents to accept a new referral without necessarily resolving the behavioral problems today. Sometimes, it may be useful to explain to the adolescent that compulsory care concerns only very limited and specific situations in psychiatry, and that we cannot force him to accept care.

**Why this is not easy?** Emergency medicine specifically cares for dependent patients and making decisions on their behalf. Using shared decision making to empower adolescents with a chronic condition is rarely used in a context of emergency. This way of thinking about psychiatric care coexists with other situations, e.g., acute psychotic episode or manic episode, where one will have to decide on behalf of the patient. Switching between this different way of thinking is not always easy.

### It Takes a Village…

**Context:** Many clinicians may be reluctant to consider psychosocial factors on behavioral problems, as they do not want to substitute for social or educative services. Consequently, it is almost certain that clinicians who feel isolated or resourceless in PED (e.g., without the additional help of a dedicated social worker or specialized nurse) will narrow their focus to the identification and treatment of psychiatric disorders.

**Principles:** Behavioral problems in adolescents stand at the cross-road of various disciplines (medicine, psychiatry, developmental psychology, pedagogy, educative and sometimes judiciary systems). As a result, the management of behavioral problems in adolescence involves the collaboration of many partners, resulting in a significant risk of role confusion between professionals and dilution of responsibilities.

- Considering their knowledge of normal development, psychopathology and care systems, psychiatrists may be at the right place to work collaboratively with a broader array of partners.
- Consultative role does not mean a substitution role, but this certainly requires clear definitions of the roles of each one. Specific meetings with other professionals outside an emergency situation may be useful to clarify this.

**Why this is not easy?** Such a collaborative role is complex, time-consuming, and requires pedagogic skills that are not always learned in medical school. Caring for patients with poor motivation to change requires a change in our traditional medical setting.

## Conclusion

Through the article we support the view that the vast majority of adolescents and their families come to the emergency room hoping that more complex and non-strictly medical or psychiatric needs will be addressed. The model presented here can be seen as a blueprint to develop an integrative, developmental, and non-judgmental view of behavioral problems in adolescence. Of note, the practical implications of this comprehensive approach will depend on the extent and availability of adolescent mental health resources locally, regionally, and nationally, both in terms of outpatient, inpatient, and various outreach modalities. While the development of such plan requires specific training, a lot of time and a large dose of collaborative functioning, this will be worthwhile if it helps clinicians to be more aware of the different dimensions involved in the behavioral problem and to feel more comfortable in assessing and treating these patients.

## Author Contributions

M-JG-B and XB: substantial contributions to the conception and design of the work. M-JG-B, SG, and UP: substantial contributions to the acquisition, analysis, or interpretation of data. M-JG-B, XB, SG, UP, and DC: drafting the work or revising it critically for important intellectual content. M-JG-B, XB, SG, UP, and DC: final approval of the version to be published. M-JG-B, XB, SG, UP, and DC: agreement to be accountable for all aspects of the work in ensuring that questions related to the accuracy or integrity of any part of the work are appropriately investigated and resolved. All authors contributed to the article and approved the submitted version.

## Conflict of Interest

The authors declare that the research was conducted in the absence of any commercial or financial relationships that could be construed as a potential conflict of interest.
